# Microwave open-ended coaxial dielectric probe: interpretation of the sensing volume re-visited

**DOI:** 10.1186/1756-6649-14-3

**Published:** 2014-06-17

**Authors:** Paul M Meaney, Andrew P Gregory, Neil R Epstein, Keith D Paulsen

**Affiliations:** 1 Thayer School of Engineering, Dartmouth College, 14 Engineering Drive, Hanover, NH 03755, USA; 2 National Physical Laboratory, Teddington, Middlesex, UK; 3 University of Calgary, Calgary, Canada

## Abstract

**Background:**

Tissue dielectric properties are specific to physiological changes and consequently have been pursued as imaging biomarkers of cancer and other pathological disorders. However, a recent study (Phys Med Biol 52:2637–2656, 2007; Phys Med Biol 52:6093–6115, 2007), which utilized open-ended dielectric probing techniques and a previously established sensing volume, reported that the dielectric property contrast may only be 10% or less between breast cancer and normal fibroglandular tissue whereas earlier data suggested ratios of 4:1 and higher may exist. Questions about the sensing volume of this probe relative to the amount of tissue interrogated raise the distinct possibility that the conclusions drawn from that study may have been over interpreted.

**Methods:**

We performed open-ended dielectric probe measurements in two-layer compositions consisting of a background liquid and a planar piece of Teflon that was translated to predetermined distances away from the probe tip to assess the degree to which the probe produced property estimates representative of the compositional averages of the dielectric properties of the two materials resident within a small sensing volume around the tip of the probe.

**Results:**

When Teflon was in contact with the probe, the measured properties were essentially those of pure Teflon whereas the properties were nearly identical to those of the intervening liquid when the Teflon was located more than 2 mm from the probe tip. However, when the Teflon was moved closer to the probe tip, the dielectric property measurements were not linearly related to the compositional fraction of the two materials, but reflected nearly 50% of those of the intervening liquid at separation distances as small as 0.2 mm, and approximately 90% of the liquid when the Teflon was located 0.5 mm from the probe tip.

**Conclusion:**

These results suggest that the measurement methods reported in the most recent breast tissue dielectric property study are not likely to return the compositional averages of the breast tissue specimens evaluated, and thus, the conclusions reached about the expected dielectric property contrast in breast cancer from this specimen study may not be correct.

## Background

Tissue dielectric properties have long been of interest to researchers because of their significant differences between tissue types [1-3]. Scientists have speculated that these properties could be harnessed for their potential to detect cancers because tumors are generally considered to have elevated water content when compared to normal tissue because of the increased hydration associated with the rapid metabolism of cancer cells and the surrounding angiogenic vasculature [4,5]. Breast cancer detection has been considered as a particularly good opportunity because the surrounding normal breast tissue of most women is dominated by adipose tissue which is well known to have low water content, and concomitantly low dielectric properties [6,7]. Early studies confirmed these expectations [7-9] but variations in the results raised questions about the data in each report. For instance, the especially low permittivity values reported in the Chaudhary study suggest that the measured tissue specimens were primarily composed of fat, and overlooked the dielectric property contributions from the normal fibroglandular breast parenchyma. A much larger and more comprehensive investigation by the Universities of Wisconsin and Calgary [10,11] assessed breast cancer contrast levels more systematically with state-of-the-art open-ended coaxial probes, and correlated the data with co-registered histopathological analyses that accounted for important contributions from factors which included the integrated fibroglandular tissue fraction. These more recent results indicate that the dielectric property contrast for breast cancer relative to a background of normal fibroglandular tissue is only a fraction of the ratios reported in previous studies. Not surprisingly, this study has influenced the direction of microwave breast imaging research, and has steered investigations towards the development of systems requiring external contrast agents [12,13] despite the fact that in vivo clinical studies are emerging which demonstrate cancer detection and monitoring to statistically significant diagnostic accuracies based on endogenous dielectric property contrast in the breast [14-17].

The development of open-ended coaxial dielectric probes during the 1980s and 1990s facilitated the routine measurement of high frequency (i.e. >100 MHz) tissue dielectric properties that were often obtained from ex-vivo specimens in the case of human tissues (because of convenience/access) [8,9,18-26], although in-vivo data from animal studies were also commonly reported [27,28]. These instruments are generally considered to be the gold standard or to provide the ground truth when characterizing a tissue’s electromagnetic properties because the tools can be validated against homogeneous samples of materials with (already) known dielectric properties. However, the sampling volume of dielectric probes, and especially how the signals (and their subsequent conversion into dielectric property estimates) from that sampling volume are influenced by small-scale property heterogeneity is critical in tissues (few are homogeneous or even reasonable approximations to the homogeneous media utilized in probe validation studies, for example, non-fatty breast tissue consists of variable patterns and percentages of interwoven adipose and fibroglandular compositions [6,9]), but is rarely considered in detail.

Hagl et al. [29] did investigate the sampling volume question and found a sensing volume of 1.5 mm (in depth) by 5 mm (in width) for a 2.2 mm diameter open-ended coaxial probe with an approach that was subsequently used to determine a sensing volume of 3 mm (in depth) by 7 mm (in width) for a 3 mm diameter dielectric probe which was applied in two large breast tissue specimen studies [10,11]. However, the experiments considered by Hagl et al. were based on homogeneous liquids in which probe tips were systematically moved to positions close to the base and side walls of a glass beaker to infer their concomitant sampling volumes (by assuming the probe’s sampling volume corresponded to the minimum volume of liquid that existed before the first evidence of signal change occurred sufficient to alter the dielectric property estimates). Unfortunately, these experiments find the minimum volume of a homogeneous liquid that is needed to measure its dielectric properties accurately, but do not determine the probe’s sampling volume, or more importantly, how the probe’s signals from the said sampling volume are influenced when the properties are not actually homogeneous. For example, if the Hagl experiments performed do approximate the sampling volume of the probe, then presumably the resultant probe property estimates from a heterogeneous sample would represent an effective average of the compositional percentages of those materials contained within the probe’s sampling volume.

In this paper, we present data from several simple experiments similar to those performed by [30], in which layered properties are used to investigate the influence of heterogeneity on the probe’s dielectric property estimates when the layers are in close proximity to the tip of the probe. While layered structures offer only one class of the infinite number of heterogeneous property distributions that exist or could be considered, they are easily controlled and simplify the problem by eliminating effects from heterogeneity in the lateral directions. The results, unfortunately, suggest that the dielectric properties are disproportionately influenced by the material resident within the first 200–400 microns of distance from the probe tip, and the probe does not behave as having a much larger sensing volume in which the resultant dielectric property estimates represent a compositional average of the dielectric properties of the materials within the volume. Numerical simulations confirm consistency between model and measurement. Even more unfortunate are the implications of these results on interpretations being made and conclusions being drawn from the data reported in [10,11]. These widely cited studies are often considered to be the definitive data on the electromagnetic properties of breast tissue/tumor, and while they do represent the largest and most systematic effort completed to date to probe the dielectric properties of breast surgical specimens, the results presented here suggest that those measurements are surface-property biased, and likely do not represent the effective dielectric properties of the volume averaged tissue that could, for example, be recovered on a cm-scale through non-invasive microwave imaging methods [14,15,17].

## Methods

### Dielectric probe measurement tank

**Figure 1:**
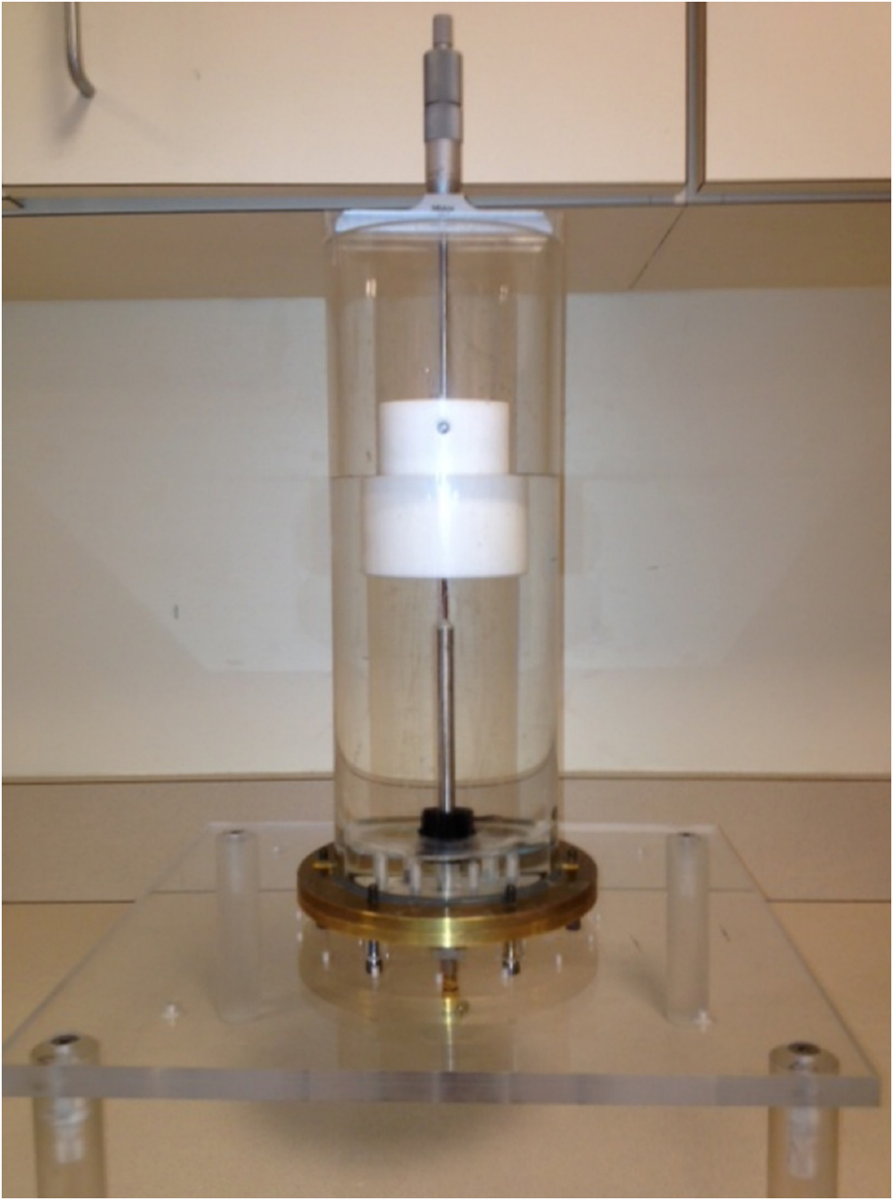
Photograph of the physical test configuration showing the cylindrical tank, dielectric probe, and coupling liquid, with the Teflon block and depth micrometer mounted to the top of the tank.

**Figure 2:**
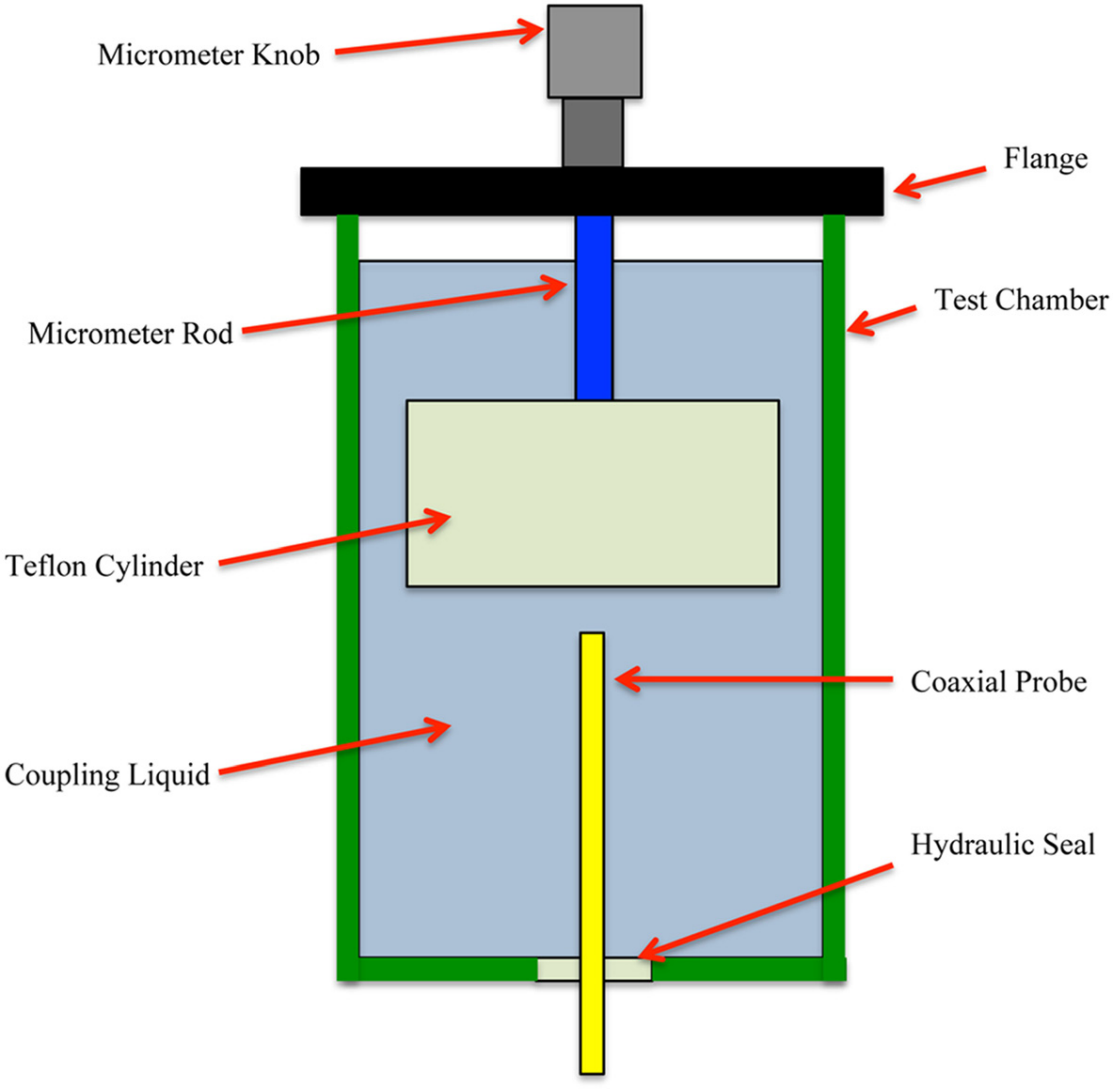
Schematic of the experimental test configuration indicating the elements of the system used to manipulate the separation distance between the dielectric probe and the Teflon cylinder.

Figure 1 shows a photograph of the actual test chamber used to conduct the measurements reported in this paper and Figure 2 shows an associated schematic diagram. It incorporated a 30.0 cm long × 10.2 cm diameter Plexiglas cylinder in which a 2.2 mm diameter Slim Form Probe from Agilent Technologies (Santa Clara, CA) was supported from below with the corresponding semi-rigid coaxial cable sliding through hydraulic seals in the base of the tank to prevent (liquid) leakage. The coaxial connector of the probe was attached to a network analyzer via a Gore PHASEFLEX OU cable (W. L. Gore & Associates, Inc., Newark, DE) which was taped down at multiple positions along its length to eliminate motion during the testing. The network analyzer was an Agilent E5071A operating from 300 KHz to 8.5 GHz (not shown). We acquired data from 100 MHz to 8.5 GHz in 100 MHz increments as a function of separation distance (between the probe tip and Teflon cylinder in Figure 1 up to 2 mm in a logarithmic fashion (e.g. more data points were acquired for Teflon positions closest to the probe that were gradually diminished as the Teflon was moved further away). We used 2 L volumes of deionized water and 0.9% clinical saline (Mediatech, Inc., Manassas, VA) as the surrounding liquid in two sets of experiments. Both liquids were kept at room temperature overnight and the network analyzer was allowed to warm up for over an hour. The typical temperature drift for the water standing in this container over a 2 hour period was 0.1°C. After calibration and measurements in water, the liquid was drained and the tank was filled with a full quantity of saline, and then drained again before the final (measured) batch of saline was added to minimize saline dilution by any remaining water. The Teflon piece was machined into a cylinder (6.2 cm diameter and 8.2 cm height) and was attached to the depth micrometer (part number 129-132, Mitutoyo Corporation, Kawasaki, Japan) post with a set screw which was placed 6 cm above the Teflon base to ensure it was sufficiently far away to not affect the probe recordings.

### Dielectric property calculations

We used the standard Agilent Dielectric Probe Kit (85070E) and the associated software package to compute the dielectric properties over a prescribed frequency range [31]. We have validated the technique in numerous different experiments. This technique is considered accurate to within 1 and 3% for the real and imaginary permittivity values, respectively, over the range of 1–12.5 GHz [32] and increase progressively for frequencies extending to 50 GHz. The measurements were calibrated with the standard procedure – in this case based on recordings from an open circuit (air), a short circuit and de-ionized water. The software essentially infers from the S-parameter data (in this case S_11_) the dielectric properties that are required to generate the associated reflection measurement. For these experiments, the shorting configuration supplied by the vendor was not convenient because of the size of the tank. Instead, a piece of aluminum foil with a soft rubber backing was used to ensure intimate contact with the probe. Benchmark measurements of Teflon, air and water were made to confirm that the probe was calibrated correctly and operating properly.

### Numerical simulations

Simulations of the fields near the probe for different measurement configurations were performed with CST Microwave Studio software (Framingham, MA) at 2 GHz and were intended to illustrate the impact of different materials on the field patterns which ultimately influence the dielectric property calculations. Dimensions of the probe and the borosilicate glass bead at the end of the open-ended coax were taken from Blackham and Pollard [33]. S_11_ values for the coaxial probe placed against a layered medium were computed through model analyses developed by Hodgetts [34] and tested in experiment by Gregory et al. [35]. This approach finds the electric fields in a geometry in which samples are bounded by a conducting cylinder. For the data presented, the size of the cylinder was chosen so that the presence of the conducting cylinder has negligible effect. It has been validated against other published methods with an error of less than 0.1%. The inversion technique to recover the complex permittivity from the S_11_ data utilized a gradient-descent method with first order differentiation developed by Grant et al. [36]. It has been validated with respect to probe dimensions over a range of dielectric properties and associated frequencies.

## Results

**Figure 3:**
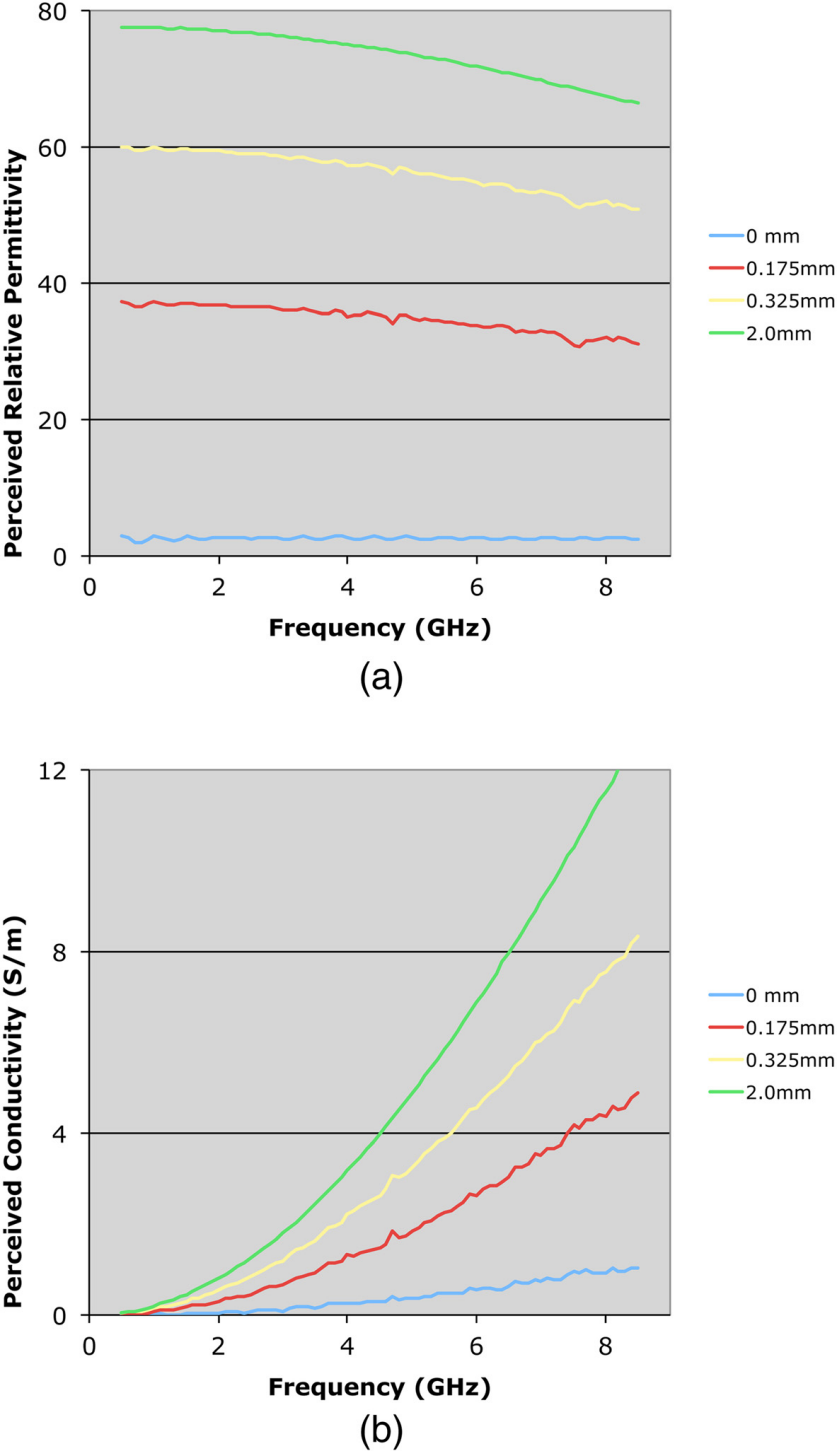
**Plots of the perceived dielectric properties as a function of frequency for four separation distances between the probe tip and Teflon cylinder: 0.0, 0.175, 0.325, and 2.0 mm, respectively. (a)** Relative permittivity, and **(b)** conductivity.

Figure 3a and b show representative plots of the perceived relative permittivity and conductivity for the Teflon cylinder positioned 0.0, 0.175, 0.325 and 2.0 mm from the probe surface, respectively, for the water coupling liquid over the 0.5 to 8.5 GHz bandwidth. In each case, three measurements were acquired and the average values are plotted. The average permittivity standard deviation (SD) for all frequencies and all water measurements was 0.34% of the mean. In terms of specific frequencies, the average permittivity SD values at 0.5 GHz and 8.5 GHz were 0.22 and 0.30%, respectively, whereas the associated maximum SDs were 0.94% and 0.99%. The average conductivity SD for all frequencies and all water measurements was 0.30% of the mean, and were 0.46% and 0.21%, respectively, at 0.5 and 8.5 GHz with maximums of 0.91% and 0.46%. Higher measurement SDs generally occurred when the probe was closest to the Teflon cylinder, as expected because these absolute property values were the lowest on the overall measurement scale, and assuming a constant absolute dielectric probe measurement error, the relative errors would be closest to their maxima. The same findings were observed in the saline measurements. Here, the average permittivity SDs for all frequencies and all 0.9% saline measurements was 0.09%, and were 0.11% and 0.11% at 0.5 GHz and 8.5 GHz, respectively, with associated maximum values of 0.19% and 0.26%. The average conductivity SDs for all frequencies and all 0.9% saline measurements was 0.11%, and were 0.15% and 0.16%, respectively, at 0.5 GHz and 8.5 GHz with associated maximum values of 0.26% and 0.27%.

**Figure 4:**
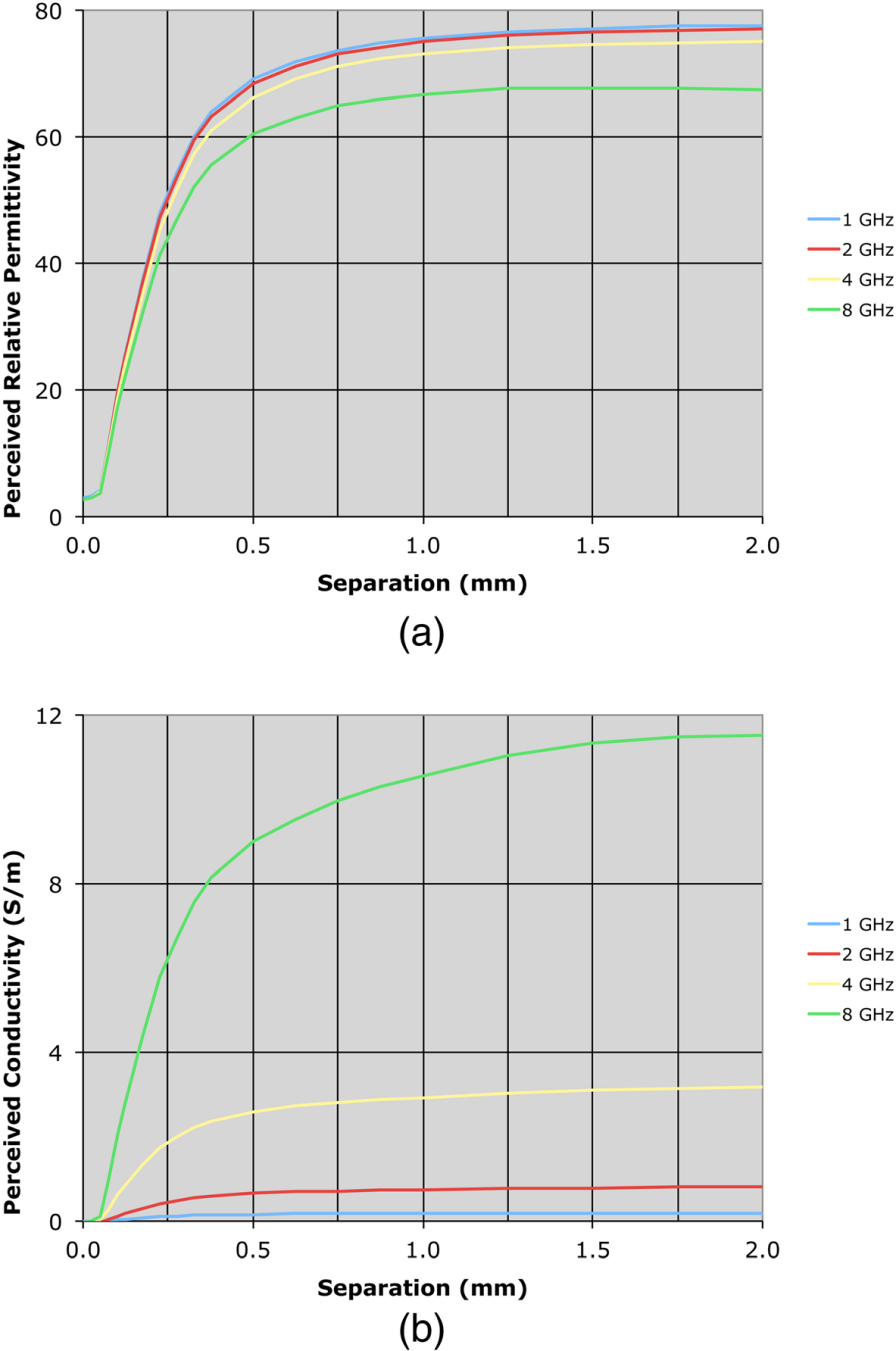
**Plots of the perceived water dielectric properties as a function of separation distance for 1, 2, 4, and 8 GHz, respectively. (a)** Relative permittivity **(b)** conductivity.

**Figure 5:**
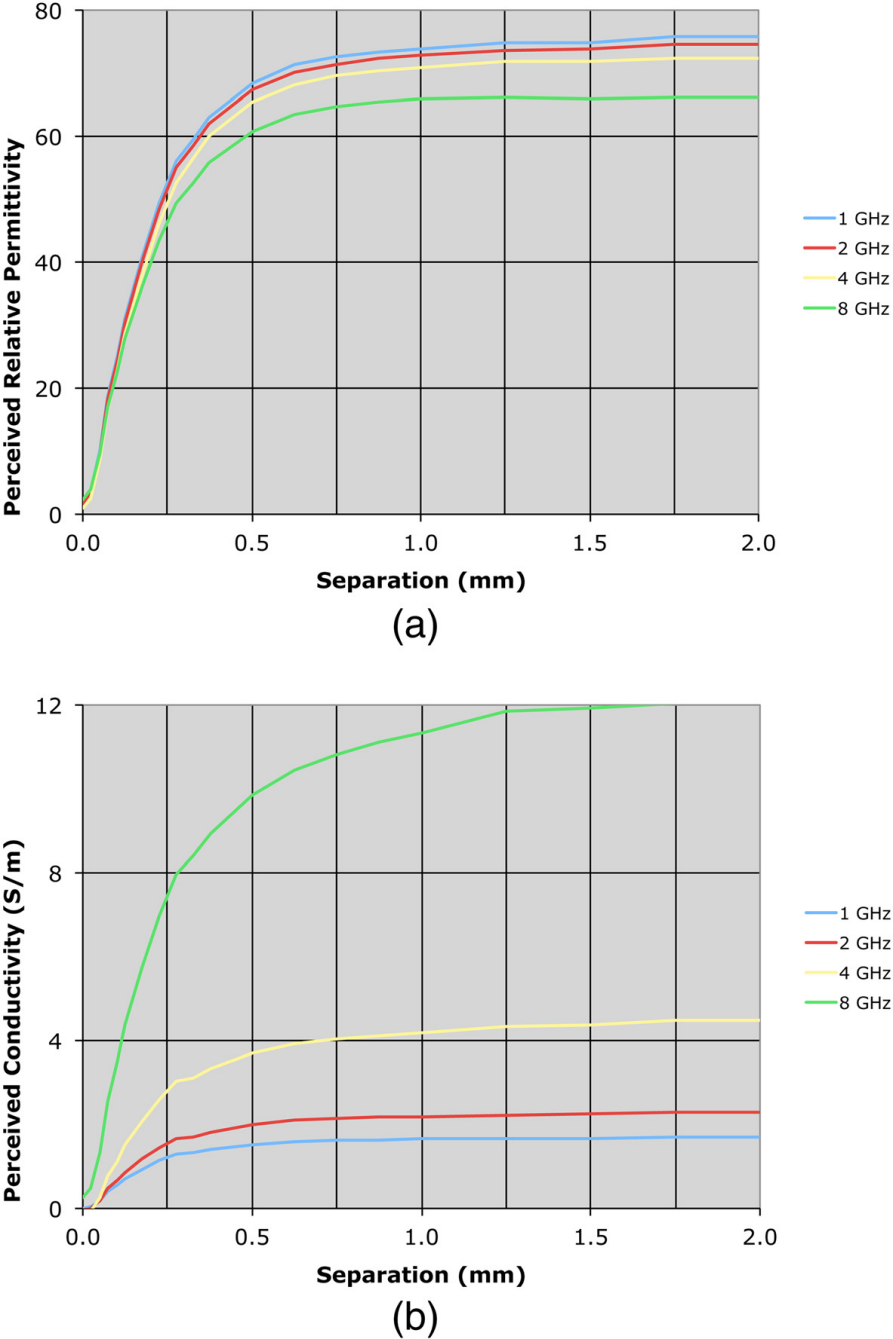
**Plots of the perceived saline dielectric properties as a function of separation distance for 1, 2, 4, and 8 GHz, respectively. (a)** Relative permittivity **(b)** conductivity.

**Figure 6:**
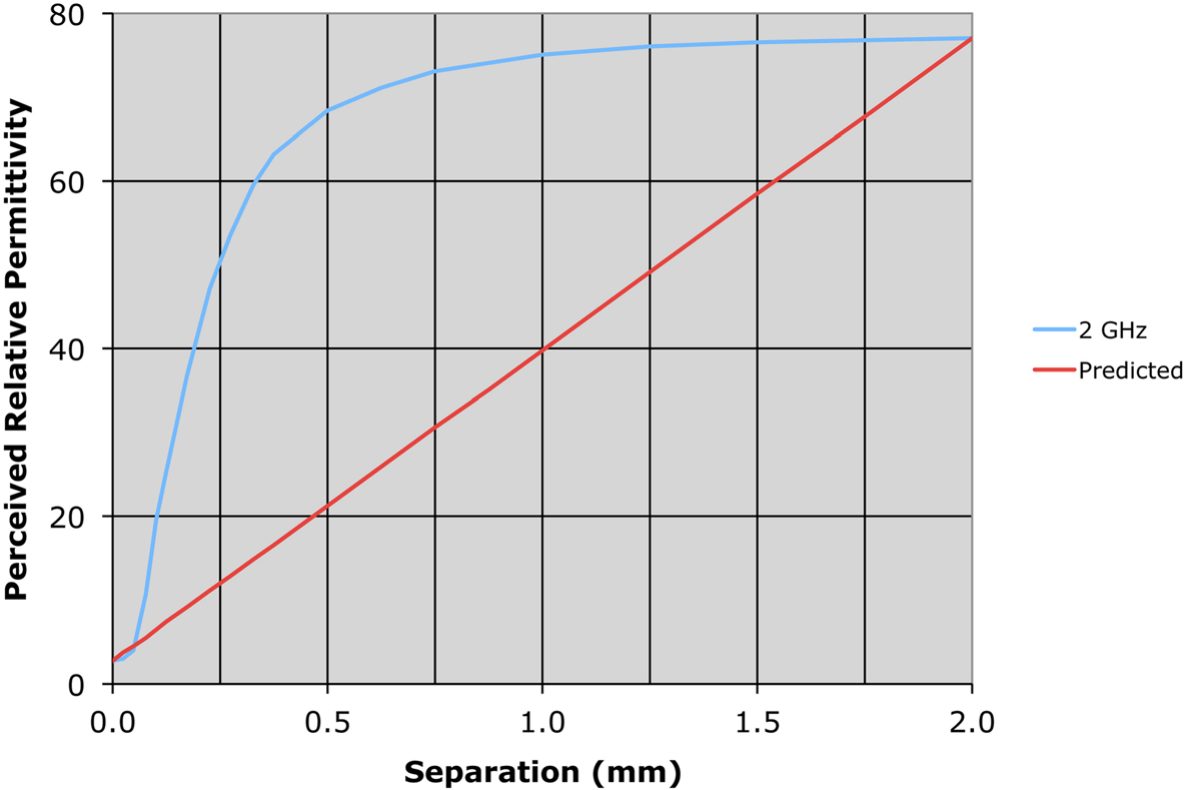
Plot of the 2 GHz perceived water relative permittivity values as a function of separation distance and the idealized curve assuming an exact average of the Teflon and water based on volume fraction within the sensing volume.

**Figure 7:**
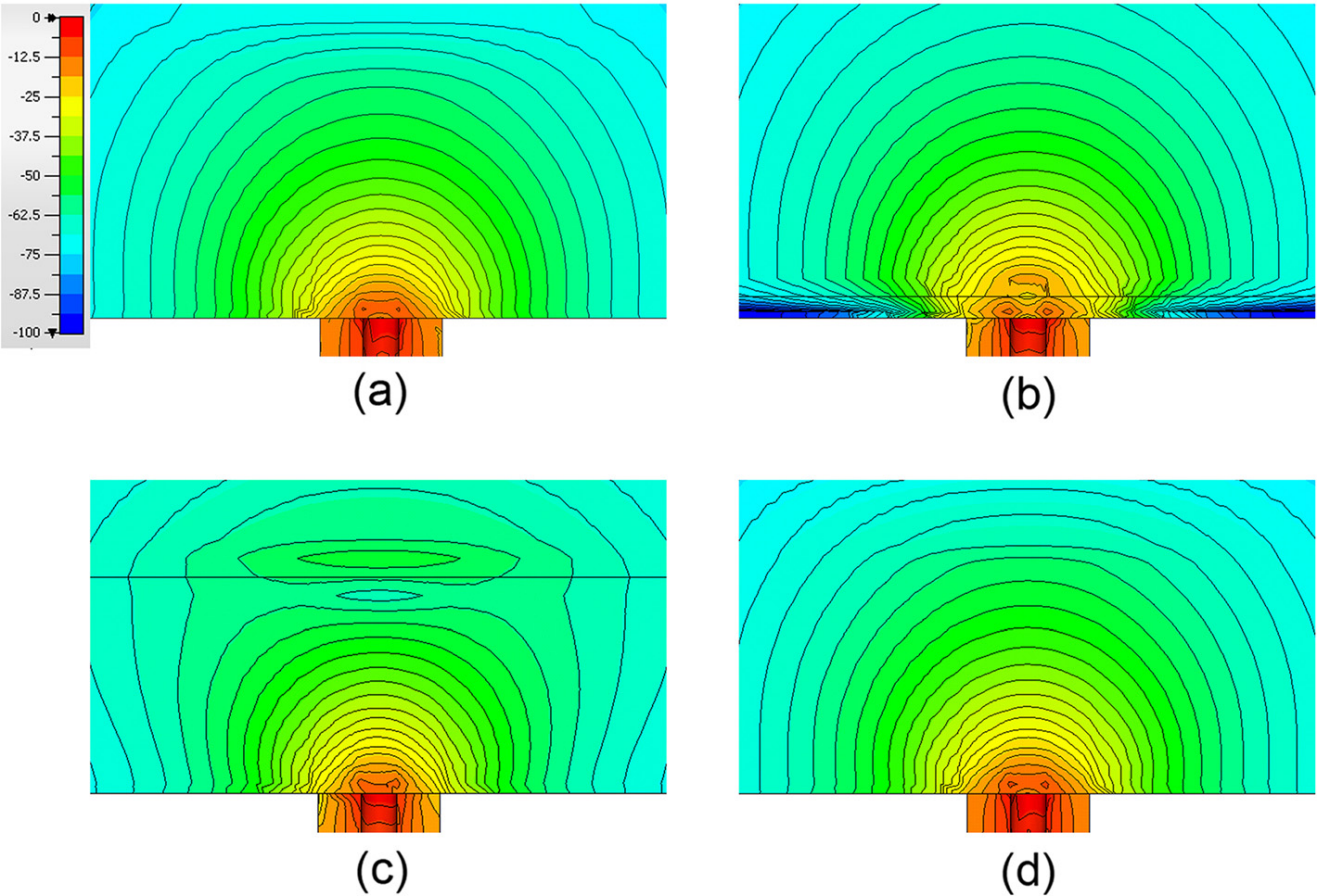
**Plots of the simulated amplitude field patterns for four different positions of the Teflon position in the water. (a)** Directly against the probe **(b)** positioned 0.3 mm from the probe **(c)** positioned 3.0 mm from the probe **(d)** submerged purely in water.

Both permittivity and conductivity plots exhibit characteristic curves that demonstrate typical dispersions over this frequency range. For the zero-distance position, the properties are effectively those of Teflon – relative permittivity of 2.0 and conductivity near zero over the band. For the 2.0 mm position, the sweeps are in line with expectations for water. The intermediate separation distance plots for 0.175 and 0.325 mm are instructive. In these cases, the properties are rapidly increasing and have diminishing influence from the Teflon such that they are approximated by a 75:25 weighted average of the water and Teflon dielectric properties at the 0.325 mm separation distance.To illustrate the nonlinear weighting of material composition in the near vicinity of the probe more clearly, we plotted the measured properties from both the water and 0.9% saline background liquids as a function of separation distance for four representative frequencies: 1.0, 2.0, 4.0 and 8.0 GHz (Figures 4 and 5). The perceived properties remain relatively flat for the first 0.05 mm of separation distance because the Teflon is compressible and we pressed the Teflon cylinder against the probe surface to ensure full contact when establishing the zero-distance separation position as the reference. Outside of this zone, relative permittivity and conductivity increase rapidly for both coupling liquids, and begin to level off after 0.5 mm of separation. The property increases appear to be more rapid for permittivity relative to conductivity, and the slopes are steeper in saline compared to the water background. The latter may occur because the extra loss in the saline limits the signal penetration to the second material. The intervening liquid clearly has the most influence within the short distances away from the probe face.As reported in some dielectric property studies, these probes are considered to have a sensing depth of 2–3 mm. Assuming the probe produces an average property estimate that is proportional to the relative composition of materials that exist over this depth, we plot the probe-measured 2 GHz water background permittivity as a function of separation distance compared to the idealized (percentage composition) relationship which is significantly different (in Figure 6).To evaluate the consistency of these results with theory, we have also computed simulated data for a representative frequency – in this case 2 GHz. The simulations in Figure 7a-d show axial field magnitude plots for four different measurement configurations: (a) the probe directly up against Teflon, (b) the probe 0.3 mm from the Teflon surface, (c) the probe 3.0 mm from the Teflon surface, and (d) the probe submerged in water without the Teflon present. The field-of-view has been cropped to the region immediately surrounding the probe tip to illustrate more clearly the field patterns nearest the probe. The presence of the Teflon impacts the overall field pattern that is observed. For instance, when the probe is 3.0 mm from the Teflon, the field pattern several centimeters away from the probe is noticeably different than when the probe is submerged purely in water (i.e., without the Teflon being present). However, minimal difference occurs in the region immediately surrounding the probe demonstrates, and the differences at more distant locations are for field strengths greater than 50 dB lower than the values closest to the probe. When the probe is only 0.3 mm from the Teflon, the field distribution is impacted to a larger degree, but the effects near the probe interface are only nominally different.

**Figure 8:**
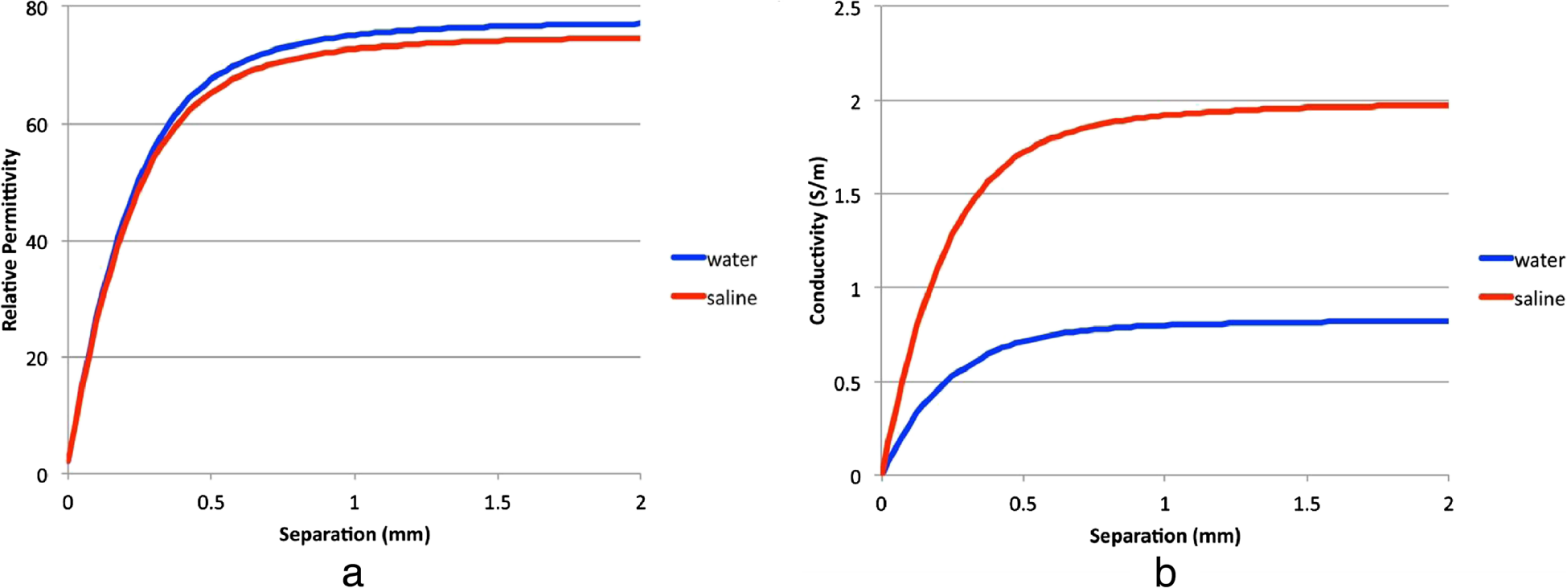
**Plots of the simulated 2 GHz perceived dielectric properties as a function of separation distance between the dielectric probe and Teflon block for both water and 0.9% saline solution coupling liquids, respectively. (a)** Relative permittivity **(b)** conductivity.

Similarly to the measurements in the previous section, simulations were performed for the probe positioned at identical spacings from the Teflon. S_11_ values were extracted from these results to compute the effective dielectric property measurements. The dielectric properties for the water and 0.9% saline solutions at 25°C were assigned to be ϵ_r_ = 73.8, σ = 0.82 S/m [32]; and ϵ_r_ = 75.0, σ = 2.0 S/m (internal measurements), respectively, whereas the effective properties of Teflon were taken as ϵ_r_ = 2.05, σ = 0.0 S/m. The probe dimensions were simulated as an outer conductor diameter of 2.2 mm, a Teflon insulator diameter of 1.7 mm, and a center conductor diameter of 0.5 mm, respectively. Figure 8a and b show the plots of the computed permittivity and conductivity as a function of separation distance between the open-ended coaxial probe and the Teflon surface. These results exhibit nearly identical behavior as the measurements in terms of property variation as a function of separation distance, which confirms that the measurement data are consistent with theory.

## Discussion

The data presented in this paper confirm the findings in Hagl et al. [29] which indicate a certain volume of material is required to achieve accurate measurements of a homogeneous sample. However, results presented here are the first to consider the dielectric property estimates obtained from an open-ended coaxial dielectric probe over microwave frequencies in the presence of a heterogeneous medium under controlled experimental conditions. Specifically, a layered volume with highly contrasting electrical properties was evaluated, and the probe recordings were consistent independently of whether the intervening layer was water or the more lossy (especially at lower frequencies) saline. The recovered properties reached 50% and 90% of those of the intervening liquid even when the fluid layer was only 0.2 and 0.5 mm thick, respectively. While the material (Teflon) at distance (from the probe) beyond the liquid layer exerted some influence, its effect was significantly diminished relative to the liquid immediately adjacent to the probe surface.

One of the challenges in deploying dielectric probes is the maintenance of contact between the probe and the material under test. Thus, they are ideal for liquid testing and soft tissue measurements because both naturally conform to the surface of the open-ended coaxial line. Accordingly, vendors such as Agilent Technologies (85070E Dielectric Probe Kit) do not recommend these probes for measuring the dielectric properties of hard materials.

## Conclusions

The implications of this report are potentially profound because the long-held presumption that an open-ended dielectric probe provides an accurate estimate of tissue properties over a heterogeneous sensing volume 2 to 3 mm below the surface of the probe is not likely to be correct. While 2–3 mm may appear to be a relatively small distance over which the dielectric probe might reasonably be expected to provide accurate property estimates, the reality is that the material within the first few hundred microns exerts the dominant influence on the estimated properties. If the open-ended coaxial probe does not recover an appropriately averaged property estimate in the layered test configuration considered here, its fidelity when used to measure the dielectric properties of more randomly arranged heterogeneous mixtures of tissue is questionable – a finding that raises serious questions about how best to utilize these probes when measuring the properties of tissues that are as heterogeneous as the breast which commonly has infiltrations of fibroglandular tissue interwoven within a matrix of adipose cells [6]. Because adipose tissue is more predominant in the breasts of many women, it is largely homogeneous and easily sampled. Fibroglandular breast tissue is more challenging, and in this respect, the results of Joines et al. [8] are particularly revealing because these investigators did attempt to separate mammary from the adipose tissues. While the Joines results only considered the frequency range from 50 to 900 MHz, their data are unambiguous in terms of demonstrating a large dielectric property contrast between malignant and mammary tissue – as much as 4:1 and 7:1 for permittivity and conductivity, respectively. These findings are in stark contrast with those of the Lazebnik et al. reports [10,11] which indicate a much smaller contrast (~10%) between malignant and fibroglandular breast tissues. While the methodology used in the Lazebnik et al. reports is sound, the results presented here indicate that the data are very likely less conclusive than is suggested in subsequent literature, and some caution is advised when interpreting these results as the basis for determining whether bulk tissue contrast (on the cm-scale) exists in the electromagnetic properties of normal versus abnormal breast tissues. Indeed, the dielectric probes used in the Lazebnik studies are not likely to return the compositional averages of the tissue specimens evaluated, and thus, the conclusions reached in the study about the expected dielectric property contrast in breast cancer may not be accurate. Accordingly, over-interpreting these results could have unintended consequences, for example, in unnecessarily steering the microwave breast imaging research community away from imaging methods based on endogenous breast tissue dielectric contrast for cancer detection. In light of the sensing volume nonlinearities of open-ended dielectric probes identified in this paper, especially when considered in the context of the positive clinical microwave breast imaging results that are emerging, caution is recommended when concluding that substantial microwave property contrast does not exist in breast cancer.

## Abbreviations

KHz: Kilohertz; MHz: Megahertz; GHz: Gigahertz; microns: Micrometers; mm: Millimeter; cm: Centimeter; L: Liter; C: Celcius; SD: Standard deviation; S-parameter: Scattering matrix parameter; S_11_: Scattering matrix parameter for a reflected signal at port 1 relative to an incident signal at port 1; ϵ_r_: Relative permittivity; σ: Electrical conductivity.

## Competing interests

Keith and Paul – MIST

Drs. Meaney and Paulsen co-founded Microwave Imaging System Technologies, Inc. (MIST) in Hanover, NH. MIST holds six patents of which three are relevant to microwave breast tomography. In addition, Drs. Meaney and Paulsen are co-inventors on two patents through Dartmouth College which are peripherally related to microwave breast tomography. There are an additional three patents pending through Dartmouth College related to microwave breast tomography. MIST won an NIH/NCI SBIR Phase I grant in 1998 and a follow-on Phase II grant in 2002. It also collaborated with the Electronics and Telecommunications Research Institute (ETRI) in Daejeon, South Korea from 2007 to 2010. Drs. Meaney and Paulsen have received consulting fees from MIST as part of the collaboration with ETRI in the past 5 years. No outside entity is sponsoring the research that went into this manuscript. It is possible that Drs. Meaney and Paulsen could benefit financially from their microwave imaging patents.

For Drs. Gregory and Epstein, there are no competing interests (financial or non-financial) related to this manuscript.

## Authors’ contributions

PMM – conceived of the idea for the study and directed both the experimental and simulation experiments. In addition, he was closely involved in most of the manuscript writing and editing. AG – performed all of the simulation experiments. He was also involved in drafting the manuscript and editing. NE – performed all of the experiments. He was also involved in drafting the manuscript. KDP – advised on all aspects of the experiments. He was involved in the manuscript writing and editing. All authors read and approved the final manuscript.

## Pre-publication history

The pre-publication history for this paper can be accessed here:

http://www.biomedcentral.com/1756-6649/14/3/prepub
